# Multilocus Sequence Analysis Reveals Three Distinct Populations of “*Candidatus* Phytoplasma palmicola” with a Specific Geographical Distribution on the African Continent

**DOI:** 10.1128/AEM.02716-18

**Published:** 2019-04-04

**Authors:** Fabian Pilet, Robert Nketsia Quaicoe, Isaac Jesuorobo Osagie, Marcos Freire, Xavier Foissac

**Affiliations:** a CIRAD, UMR PVBMT, Saint-Pierre, La Réunion, France; b Council for Scientific Research Program–Oil Palm Research Institute (CSIR-OPRI), Sekondi, Ghana; c Nigerian Institute for Oil Palm Research (NIFOR), Benin City, Nigeria; d Instituto de Investigaçao Agraria de Moçambique (IIAM), Maputo, Mozambique; e UMR1332 Biologie du Fruit et Pathologie, INRA, Université de Bordeaux, Villenave-d’Ornon, France; University of Georgia

**Keywords:** phytoplasma, coconut lethal yellowing disease, multilocus sequence typing

## Abstract

To sustain epidemiological studies on coconut lethal yellowing disease (CLYD), a devastating disease in Africa caused by a phytoplasma, we developed a multilocus sequence typing (MLST) scheme for “*Candidatus* Phytoplasma palmicola” based on eight housekeeping genes. At the continental level, eight different sequence types were identified among 132 “*Candidatus* Phytoplasma palmicola”-infected coconuts collected in Ghana, Nigeria, and Mozambique, where CLYD epidemics are still very active. “*Candidatus* Phytoplasma palmicola” appeared to be a bacterium that is subject to strong bottlenecks, reducing the fixation of positively selected beneficial mutations into the bacterial population. This phenomenon, as well as a limited plant host range, might explain the observed country-specific distribution of the eight haplotypes. As an alternative means to increase fitness, bacteria can also undergo genetic exchange; however, no evidence for such recombination events was found for “*Candidatus* Phytoplasma palmicola.” The implications for CLYD epidemiology and prophylactic control are discussed. The usefulness of seven housekeeping genes to investigate the genetic diversity in the genus “*Candidatus* Phytoplasma” is underlined.

**IMPORTANCE** Coconut is an important crop for both industry and small stakeholders in many intertropical countries. Phytoplasma-associated lethal yellowing-like diseases have become one of the major pests that limit coconut cultivation as they have emerged in different parts of the world. We developed a multilocus sequence typing scheme (MLST) for tracking epidemics of “*Ca*. Phytoplasma palmicola,” which is responsible for coconut lethal yellowing disease (CLYD) on the African continent. MLST analysis applied to diseased coconut samples collected in western and eastern African countries also showed the existence of three distinct populations of “*Ca*. Phytoplasma palmicola” with low intrapopulation diversity. The reasons for the observed strong geographic patterns remain to be established but could result from the lethality of CLYD and the dominance of short-distance insect-mediated transmission.

## Abstract (precis)

Coconut is an important crop for both industry and small stakeholders in many intertropical countries. Phytoplasma-associated lethal yellowing-like diseases have become one of the major pests that limit coconut cultivation as they have emerged in different parts of the world. We developed a multilocus sequence typing scheme (MLST) for tracking epidemics of “*Ca*. Phytoplasma palmicola,” which is responsible for coconut lethal yellowing disease (CLYD) on the African continent. MLST analysis applied to diseased coconut samples collected in western and eastern African countries also showed the existence of three distinct populations of “*Ca*. Phytoplasma palmicola” with low intrapopulation diversity. The reasons for the observed strong geographic patterns remain to be established but could result from the lethality of CLYD and the dominance of short-distance insect-mediated transmission.

## INTRODUCTION

Molecular epidemiology requires easy-to-use tools that can sufficiently discriminate at a population level. Both multilocus sequence typing (MLST) and multilocus variable number tandem repeat (VNTR) analysis (MLVA) have been developed to fulfill this objective and then were applied to the surveillance of human, animal, and plant pathogens (1, 2). Next-generation sequencing (NGS) allows the study of bacterial diversity at the genomic level through whole-genome sequencing (WGS). WGS considerably increased the resolution of subpopulations of Staphylococcus aureus (3), Mycobacterium abscessus (4), and Mycoplasma pneumoniae (5). Mycoplasma-like phytoplasmas are bacteria of the class *Mollicutes* (6). Mycoplasmas are restricted to vertebrate hosts, while phytoplasmas are plant pathogens vectored through circulative-propagative transmission by phloem-feeding insects of the order Hemiptera (7, 8).

The inability to grow phytoplasmas in axenic culture remains a major constraint in developing WGS strategies, as it limits access to adequate amounts of pure phytoplasma DNA. Only six full-chromosome sequences of phytoplasmas have been deciphered to date (9), and genome surveys depend on fastidious and complex molecular strategies (10–13). However, metagenomic approaches through NGS have opened new opportunities, and 17 additional phytoplasma genome draft sequences have been deposited in GenBank (NCBI). Such genome-wide sequence analyses have provided easy access to genetic markers, allowed unbiased definitions of species boundaries in the genus “*Candidatus* Phytoplasma,” and have revealed the horizontal transfer of potential mobile units and effectors (14, 15).

MLST schemes have been developed for many *Mollicutes*, almost exclusively *Mycoplasma* and *Ureaplasma*, because of their impacts on humans and animals (16–18). For phytoplasmas, most of the MLST schemes that have been developed for “*Candidatus* Phytoplasma” species or 16S taxonomic groups target rRNAs, housekeeping genes, protein coding genes, and also positively selected genes involved in the interaction with the host (19, 20). A single study following the classical requirement of an MLST scheme that includes at least seven housekeeping genes and targets a small number of samples is currently available (2, 21). Other studies that targeted three to five housekeeping genes have addressed taxonomic or epidemiological questions (22, 23). Nevertheless, primers tend to be species specific most of the time, and MLST schemes are not transferable to other phytoplasma species or taxonomic groups.

Among the numerous diseases caused by phytoplasmas, coconut lethal yellowing diseases (CLYD), also known as lethal yellowing type syndromes (LYTS), are among the most destructive, with significant economic and social impact (24). CLYD are present in Africa, the Caribbean, Central America, and Oceania, where they are associated with different “*Ca*. Phytoplasma” species according to the area considered (25).

In Africa, CLYD are responsible for the loss of millions of coconut trees since the beginning of the 20th century. In east Africa, CLYD were first described in 1905 in Tanzania (26) and then in Kenya (27). The first description in Mozambique was in 1958 in Cabo Delgado (28), and that in Zambezia was in 1972 (29), growing to an epidemic in the 1990s. In West Africa, CLYD was described for the first time in Nigeria as Awka wilt disease in 1917 (30). From the 1930s, similar diseases were described in the eastern Volta region of Ghana (31), in Togo (32), and in Cameroon (33). In 1964, coconut lethal yellowing was observed in the western region of Ghana (31) and in the 2000s in Ivory Coast (34). Molecular characterization of the phytoplasmas associated with these different syndromes was performed only in a few countries (see Fig. S1 in the supplemental material), and two different “*Candidatus* Phytoplasma” species described exclusively on the African continent were identified: “*Ca*. Phytoplasma cocostanzania” in Kenya and Tanzania (35) and North Mozambique (36) and “*Ca*. Phytoplasma palmicola” in Ghana, Nigeria (37), Ivory Coast (38), and Mozambique (39).

Based on the sequence and restriction fragment length polymorphism (RFLP) pattern of the central part of its 16S rRNA gene, “*Ca*. Phytoplasma palmicola” is classified in group 16SrXXII (40) and is divided into two subgroups that can be differentiated by HaeIII enzymatic restriction. Subgroup 16SrXXII-A includes “*Ca*. Phytoplasma palmicola” phytoplasmas from Mozambique and Nigeria, and subgroup 16SrXXII-B corresponds to “*Ca*. Phytoplasma palmicola” from Ghana and Ivory Coast (37). A few studies have been conducted to differentiate between populations of “*Ca*. Phytoplasma palmicola” at the national level by using different genes. The sequence of the *secA* gene differentiated between “*Ca*. Phytoplasma palmicola” samples from Ivory Coast, Ghana, and Mozambique (41, 42), while the *rplV* gene was able to distinguish “*Ca*. Phytoplasma palmicola” isolates from different Ghanaian regions (43).

The propagation of “*Ca*. Phytoplasma palmicola” depends on its insect vectors, dissemination potential, alternative host plants, and a still unproved seed transmission. In practice, sampling CLYD and insects potentially vectoring the disease is also challenging due to the difficulty in accessing the several-meters-high canopy of coconut trees. Despite years of epidemiological research, the insect vectors are still unidentified. The rationale of this study is based on the assumption that deciphering the genetic structure of “*Ca*. Phytoplasma palmicola” populations at regional, national, and continental levels and mapping the distribution of its genetic variants will give insights into the origin and pathways of the spread of “*Ca*. Phytoplasma palmicola.” We therefore developed an eight-gene MLST scheme that follows the general requirements for genotyping bacterial agents and applied this scheme to a set of 132 “*Ca*. Phytoplasma palmicola” isolates collected from three African countries where active CLYD epidemics had been previously associated with the two “*Ca*. Phytoplasma palmicola” taxonomic subgroups, 16SrXXII-A and 16SrXXII-B.

## RESULTS

### Housekeeping gene selection and properties.

Eight housekeeping genes, namely, *dnaC*, *gyrB*, *leuS*, *lpd*, *secA*, *recA*, *rsmI*, and *rplV*, were selected to investigate the genetic diversity among 132 samples of “*Ca*. Phytoplasma palmicola” originating from three of the main African countries affected by this phytoplasma, i.e., Ghana, Nigeria, and Mozambique. The primers designed for this study successfully amplified the respective gene targets for all 132 DNA samples of “*Ca*. Phytoplasma palmicola” of both the 16SrXXII-A and 16SrXXII-B groups, irrespective of their collection dates or geographic origins. The PCR products observed on agarose gels each showed a unique and clear DNA band from 553 to 983 bp depending on the target gene (Table 1). None of the 1,056 double-strand-sequenced PCR products displayed double peaks or ambiguous bases, thus demonstrating the specificity of the primers and the absence of mixed infections.

**TABLE 1:** PCR primer pairs used to amplify each “*Ca*. Phytoplasma palmicola” MLST locus

Target gene	Primer	Sequence (5′ to 3′)	*T_a_* (°C)^*a*^	Amplicon (bp)^*b*^	Reference or source
*lpd*	lpd_CPpml-F	AGTTCAATTAGATGTTTGTCCTCGT	58	833	This study
	lpd_CPpml-R	TCAGATAAAGTTGGATGAGGATGA			
*dnaC*	dnaC_CPpml-F	CTGCTCGTCCTTCTATGGGA	58	670	This study
	dnaC_CPpml-R	AGCCACAATTAATTCTATATTACCTG			
*leuS*	leuS_CPpml-F	CAGAACAATATGCTTTACAAACAGG	58	783	This study
	leuS_CPpml-R	TCACAAGCAGGAACAGACATAA			
*gyrB*	gyrB_CPpml-F	TGGAAAAATGTTTGTTAGCTGT	54	732	This study
	gyrB_CPpml-R	CGAGCAGTTACTTCTTCGCC			
*secA*	secA_CPpml-F	AAAAACCTCAAACCACAACATT	54	750	This study
	secA_CPpml-R	TATCAGTACCACGACCAGCC			
*rsmI*	rsmI_CPpml-F	ATATATCAGATATTAGTTTTCGAGCT	54	553	This study
	rsmI_CPpml-R	TTCACCATGAATAATAGTTTCGAA			
*recA*	recA_CPpml-F	TTCCCACTGGTTCTTTGTCTTT	54	832	This study
	recA_CPpml-R	ATCAGCTATGTTTGGGTTTTGT			
*rplV* and *rpsC*	rpLYF1	TTTAAAGAAGGTATTAACATGA	51	983	50
	rpLYR1	TAATACCTATAACTCCGTG			
16S rRNA gene	P1	AAGAGTTTGATCCTGGCTCAGGATT	56	1,756	48
	P7	CAGAACAATATGCTTTACAAACAGG			

a *T_a_*, annealing temperature used in PCRs.

b PCR product length in base pairs observed by electrophoresis on agarose gel.

The GC content of “*Ca*. Phytoplasma palmicola” housekeeping genes (cGC) was between 26.3% (*gyrB*) and 30.4% (*recA*) (Table 2). The average cGC for the eight genes was 27.80%.

**TABLE 2:** Genetic parameters calculated for each individual locus and concatenated sequences from the “*Ca*. Phytoplasma palmicola” MLST scheme^*a*^

Parameter	Population	*n*	Value for:								
			*lpd*	*dnaC*	*leuS*	*gyrB*	*secA*	*rsmI*	*recA*	*rplV*	Concat
Length (bp)			630	543	657	606	627	417	708	324	4,512
cG+C (%)			29.0	26.7	27.3	26.3	26.5	27.8	30.4	28.3	27.8
*K_a_*/*K_s_*			0.172	0.082	0.103	0.010	0.168	0.067	0.037	0.077	0.132
H/ST (SS)	GHA	96	3 (2)	1 (0)	2 (2)	1 (0)	1 (0)	2 (1)	1 (0)	2 (1)	4 (6)
	NGA	4	1 (0)	1 (0)	1 (0)	1 (0)	1 (0)	1 (0)	1 (0)	1 (0)	1 (0)
	MOZ	32	2 (1)	2 (1)	2 (1)	2 (1)	1 (0)	2 (3)	2 (3)	2 (1)	3 (9)
	AFR	132	6 (36)	4 (20)	5 (28)	4 (25)	3 (23)	5 (23)	4 (36)	5 (14)	8 (205)
π	GHA	96	0.0008	0.00	0.0010	0.00	0.00	0.0005	0.00	0.0010	0.0004
	MOZ	32	0.0001	0.0001	0.0008	0.0001	0.00	0.0002	0.0003	0.0002	0.0002
	AFR	132	0.0213	0.0114	0.0153	0.0147	0.0117	0.0195	0.0167	0.0112	0.0154
Hd	GHA	96	0.455		0.321			0.189		0.321	0.475
	MOZ	32	0.063	0.063	0.516	0.063		0.063	0.063	0.063	0.546
	AFR	132	0.658	0.418	0.613	0.418	0.415	0.518	0.418	0.587	0.696
Tajima's D	GHA	96	0.4105		1.0039			–0.0362		0.7491	0.9207
	MOZ	32	–1.1424	–1.1424	1.6467	–1.1424		–1.1424	–1.7295	–1.1424	–1.6703
	AFR	132	2.8042^*c*^	1.9326	2.6036^*b*^	2.3302^*b*^	2.1193^*b*^	2.6459^*b*^	2.0925^*b*^	1.1031	2.5782^*b*^
Fu & Li's D	GHA	96	0.6886	0.0	0.6886	0.00	0.00	0.4949	0.00	0.4949	1.1233
	MOZ	32	–1.7034	–1.7034	0.5871	–1.7036	0.00	–1.7034	–2.7326^*b*^	–1.7034	–3.3707^*c*^
	AFR	132	2.1032^*c*^	1.7462^*c*^	1.9567^*c*^	1.9168^*c*^	1.8261^*c*^	1.8261^*c*^	1.2041	0.9461	2.4606^*c*^
Fu & Li's F	GHA	96	0.7049	0.00	0.9183	0.00	0.00	0.3910	0.00	0.6651	1.2465
	MOZ	32	–1.7820	–1.7820	1.0149	–1.7034	0.00	–1.7820	–2.8304	–1.7820	–3.3301
	AFR	132	2.8710	2.1773	2.6581	2.4961	2.3260	2.5722	1.8837	1.1993	3.0119

a Parameters were determined for Ghanaian (GHA), Nigerian (NGA), and Mozambican (MOZ) populations and at the African continent (AFR) level. The lengths of the trimmed gene and concatenated sequences (Concat) in base pairs considered for MLSA and their coding G+C percentages (cG+C) are presented. The synonymous/nonsynonymous substitution (*K_a_*/*K_s_*) ratio was calculated using the MEGA 7.0 program. The number of haplotypes (H) or sequence types (ST) with the number of segregating sites (SS), haplotype diversity (Hd), and nucleotide diversity (π) were calculated using DnaSP 6.1, as well as the neutrality tests using Tajima's D, Fu and Li's D, and Fu and Li's F statistics. Most parameters were not calculated for the Nigerian population (NGA) because of the occurrence of a single haplotype.

b *P* < 0.05.

c *P* < 0.02.

The *K_a_*/*K_s_* values, measuring the ratio of the number of nonsynonymous substitutions per nonsynonymous site (*K_a_*) to the number of synonymous substitutions per synonymous site (*K_s_*), are presented in Table 2. Low *K_a_*/*K_s_* values ranging from 0.01 (*gyrB*) to 0.17 (*lpd*) indicated that all the selected genes underwent purifying selection.

### Genetic diversity of “*Ca*. Phytoplasma palmicola.”

The number of haplotypes (H/ST), the number of segregating sites (SS), the nucleotide diversity (π), and the haplotype diversity (Hd) for each housekeeping gene and its concatenated sequence are presented in Table 2 according to the infected-coconut country of origin.

The less discriminating gene, *secA*, revealed three haplotypes, H*_secA_*1 to H*_secA_*3, each corresponding to a different country of origin. The more discriminating gene, *lpd*, differentiated into six haplotypes, H*_lpd_*1 to H*_lpd_*6.

**FIG 1:**
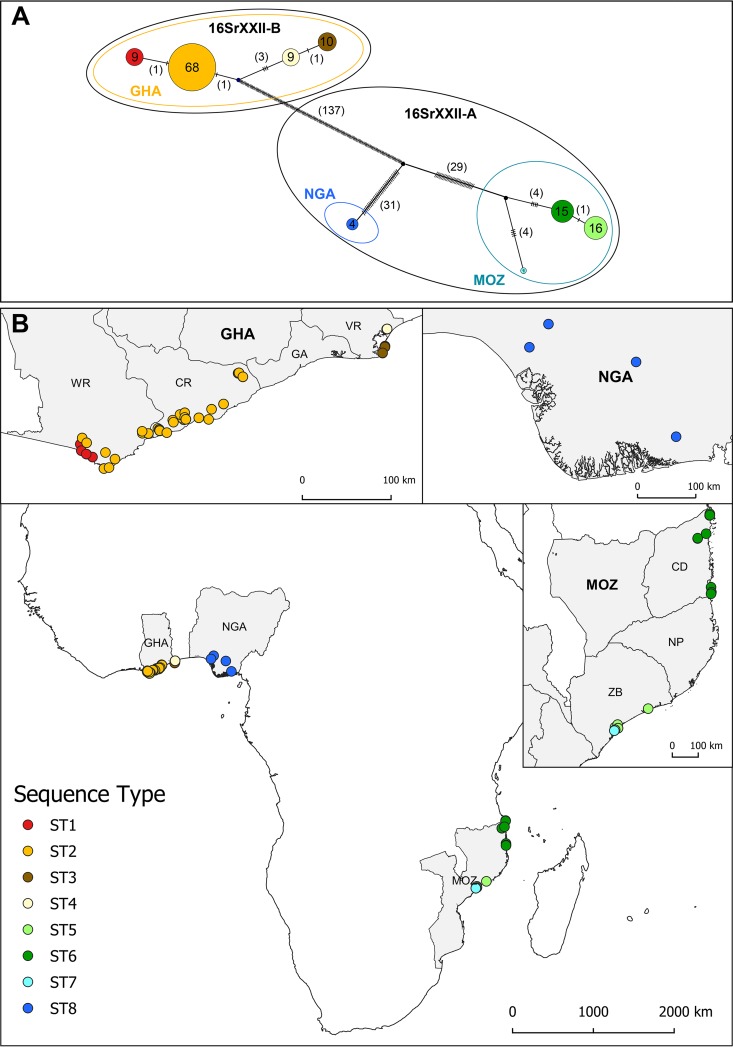
(A) Integer neighbor-joining network calculated from eight housekeeping gene (*dnaC, gyrB, leuS, lpd, recA, rplV, rsmI, secA*) concatenated sequences (4,512 bp) of 132 “*Candidatus* Phytoplasma palmicola” samples originating from three African countries, Ghana (GHA), Mozambique (MOZ), and Nigeria (NGA). Each circle represents a sequence type (ST). The numbers inside each circle correspond to the numbers of samples presenting identical STs, and the numbers in parentheses represent the numbers of single-nucleotide polymorphism (SNPs) between STs. Small ellipses describe the three different geographic populations. Large ellipses represent the 16Sr RFLP subgroup deduced from 16S rRNA gene sequences. (B) Geographical distribution of the 132 “*Ca*. Phytoplasma palmicola” samples according to their STs at the African continent level, in Ghana (96 samples), in Mozambique (32 samples), and in Nigeria (4 samples). The Ghanaian coastal regions of western region (WR), central region (CR), greater Accra (GA), and Volta region (VR) and the Mozambican provinces of Cabo Delgado (CD), Nampula (NP), and Zambezia (ZB) are delimited.

The eight genes were concatenated to obtain a sequence of 4,512 bp for each isolate. With 205 segregating nucleotides, the concatenated sequences allowed the differentiation of eight sequence types (STs) (Table 3; Fig. 1). This combination of housekeeping gene haplotypes showed that four STs are present in Ghana (ST1 to ST4), three different STs in Mozambique (ST5 to ST7), and a unique ST, ST8, in Nigeria (Table 3; Fig. 1). All but ST7 were represented by several isolates.

**TABLE 3:** Combination of the 8 housekeeping gene haplotypes allowing discrimination of the 8 STs of “*Ca*. Phytoplasma palmicola”

Sequence type	Geographic population	*n*	Housekeeping gene haplotype							
			H *lpd*	H *dnaC*	H *leuS*	H *gyrB*	H *secA*	H *rsmI*	H *recA*	H *rplV*
ST1	Ghana	9	H*_lpd_*1	H*_dnaC_*1	H*_leuS_*1	H*_gyrB_*1	H*_secA_*1	H*_rsmI_*1	H*_recA_*1	H*_rplV_*1
ST2	Ghana	68	H*_lpd_*2	H*_dnaC_*1	H*_leuS_*1	H*_gyrB_*1	H*_secA_*1	H_rsmI_1	H*_recA_*1	H*_rplV_*1
ST3	Ghana	10	H*_lpd_*3	H*_dnaC_*1	H*_leuS_*2	H*_gyrB_*1	H*_secA_*1	H*_rsmI_*2	H*_recA_*1	H*_rplV_*2
ST4	Ghana	9	H*_lpd_*3	H*_dnaC_*1	H*_leuS_*2	H*_gyrB_*1	H*_secA_*1	H*_rsmI_*1	H*_recA_*1	H*_rplV_*2
ST5	Mozambique	16	H*_lpd_*4	H*_dnaC_*2	H*_leuS_*3	H*_gyrB_*2	H*_secA_*2	H*_rsmI_*3	H*_recA_*2	H*_rplV_*3
ST6	Mozambique	15	H*_lpd_*4	H*_dnaC_*2	H*_leuS_*4	H*_gyrB_*2	H*_secA_*2	H*_rsmI_*3	H*_recA_*2	H*_rplV_*3
ST7	Mozambique	1	H*_lpd_*5	H*_dnaC_*3	H*_leuS_*4	H*_gyrB_*3	H*_secA_*2	H*_rsmI_*4	H*_recA_*3	H*_rplV_*4
ST8	Nigeria	4	H*_lpd_*6	H*_dnaC_*4	H*_leuS_*5	H*_gyrB_*4	H*_secA_*3	H*_rsmI_*5	H*_recA_*4	H*_rplV_*5

The 16S rRNA of 41 samples representing each of the eight STs was sequenced (6 isolates of ST1, 9 of ST2, 4 of ST3, 5 of ST4, 6 of ST5, 6 of ST6, 1 of ST7, 4 of ST8). Two types of sequences were identified. Seventeen samples corresponding to ST5 to ST8 originating from Nigeria and Mozambique were all identical to the 16S rRNA sequence of the LDN strain from Nigeria (accession number Y14175) (44, 45) and were therefore classified as members of subgroup 16SrXXII-A. Twenty-four samples of ST1 to ST4 from Ghana were all identical to the 16S rRNA of CSPW-dna19 and were therefore assigned to subgroup 16SrXXII-B (accession number KF364359) (37). Sequences from subgroups 16SrXXII-A and -B differed by six single-nucleotide polymorphisms (SNPs) over 1,505 bp (99.6% homology).

### Geographic distribution of the “*Ca*. Phytoplasma palmicola” sequence types.

All the DNA samples were spatially referenced, allowing the mapping of the distribution of their corresponding STs. The geographic distribution of the STs illustrates their aggregation in each country (Fig. 1B). Each ST presents a continuous distribution, each constituting a well-defined focus, except for ST7, which was represented by the single isolate MZ12-187. The geographic origins of the 132 samples and their corresponding STs are available (see Data Set S1 in the supplemental material). In central Ghana (CR), ST2 was the most abundant and only type present, while in the western region (WR), it coexisted with ST1, a single SNP variant of ST2 (Fig. 1). ST3 and ST4 constituted two foci in the eastern Volta region of Ghana (VR). In Nigeria, the second western African country surveyed, only ST8 was found, but only four DNA samples were analyzed. In Mozambique in East Africa, ST5 and ST6 were prevalent. ST5 was restricted to the province Zambezia, while ST6 appeared only along the coast of the northern province Cabo Delgado. A unique ST7 sample was also localized to Zambezia. ST7 exhibited 8 or 9 SNPs compared to the two other Mozambican sequence types, ST5 and ST6 (Fig. 1A).

### Population structure of “*Ca*. Phytoplasma palmicola.”

Tajima's D and Fu and Li's D and F were calculated for each housekeeping gene, and the concatenated sequences were calculated for each country and at the African continent level (Table 2). Tajima's D was positive but not significant for the concatenated sequences and sequences of all the genes except *rsmI* from Ghana. Even though they were not significant, the calculated Tajima's D values were negative using most of the Mozambican sequences, probably because ST7 was represented by a unique sample. With the exception of *dnaC* and *rplV*, all Tajima's D and Fu and Li's D and F values were significantly positive at the continental level, suggesting bottleneck effects.

An integer neighbor-joining network was calculated using the concatenated sequences of the 132 isolates (Fig. 1A). Of over 205 segregating sites, 202 were parsimony informative. The structure of the network illustrates both strong regional differentiation and the clonal structure of the “*Ca*. Phytoplasma palmicola” populations. From the network, we observed three distinct populations. Multilocus sequence analysis (MLSA)-concatenated sequences of samples from Ghana are similarly distant from the closer Nigerian and Mozambican genotypes, from which they differed by 169 (3.75%) and 171 (3.79%) SNPs, respectively. While from the same 16SrXXII-A subgroup, Mozambican and Nigerian samples differed by at least 64 SNPs (1.42%). The within-country genetic diversity was low, with no SNPs for Nigeria, 6 SNPs for Ghana, and 9 SNPs for Mozambique.

Within the 16Sr subgroups, the genetic diversity was higher for subgroup 16SrXXII-A than for subgroup 16SrXXII-B. In detail, the STs of subgroup 16srXXII-B split into two divergent branches (Fig. 1A). Only 1 SNP differentiated STs within the same subregion, whereas interregional diversity ranged from 4 to 6 SNPs. Within subgroup 16SrXXII-A, the two main STs, ST5 and ST6, differed by only one mutation, while ST7 differed by eight mutations compared to the more closely related ST6 in Mozambique. In this subgroup, the Nigerian ST8 differed from the Mozambican STs by a minimum of 64 SNPs but was, however, more distant from the Ghanaian STs of the other subgroup by a minimum of 138 SNPs.

### Absence of recombination among “*Ca*. Phytoplasma palmicola” sequence types.

Split networks were calculated from “*Ca*. Phytoplasma palmicola” STs for each housekeeping gene (Fig. S2). The split-graph structures observed for *lpd* and *rsmI* did not appear to exclude recombination phenomena, and phi test results with *P* values of 0.3405 and 0.1417, respectively, were not significant irrespective of the gene being considered. The phi test result calculated for the concatenated sequences was also not significant (*P* = 0.0748), and the Recombination Detection Program (RDP; http://web.cbio.uct.ac.za/~darren/rdp.html) did not identify any recombination signals.

### Interspecific comparison using the developed scheme.

**FIG 2:**
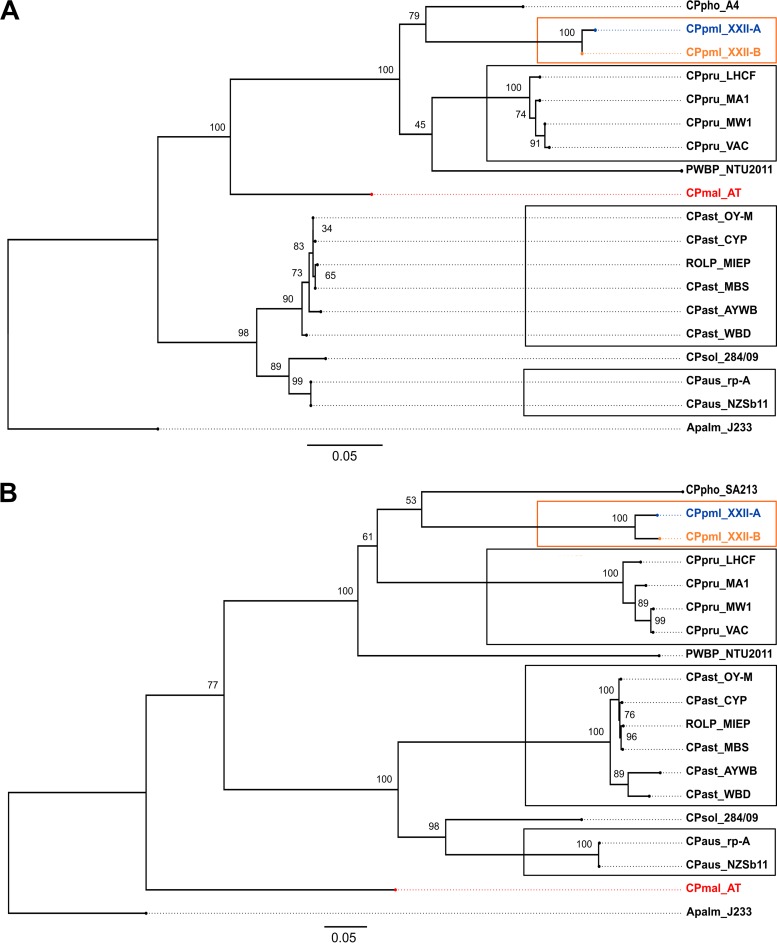
Phylogenetic relationship of 18 *Candidatus* phytoplasmas. A rooted maximum likelihood tree was calculated for the 16S rRNA gene sequences (A) and for 3,756-bp concatenated DNA sequences of the *dnaC, leuS, gyrB, rsmI, lpd, secA*, and *rplV* housekeeping genes with the recombinant region removed (B). The codes used are as follows: CPmal_AT (“*Ca*. Phytoplasma mali” strain AT [CU469464.1]), CPaus_rp-A (“*Ca*. Phytoplasma australiense” [NC_010544.1]), CPast_OY-M (“*Ca*. Phytoplasma asteris” strain onion yellows phytoplasma OY-M [NC_005303.2]), CPpru_MW1 (“*Ca*. Phytoplasma pruni” strain milkweed yellows-MW1 [AKIL00000000.1]). CPpru_MA1 (“*Ca*. Phytoplasma pruni” strain Italian clover phyllody MA1 [AKIM00000000.1]), CPpru_VAC (“*Ca*. Phytoplasma pruni” strain vaccinium witches'-broom VAC [AKIN00000000.1]), PWBP_NTU2011 (peanut witches'-broom phytoplasma NTU2011 [AMWZ00000000.1]), CPaus_NZSb11 (“*Ca*. Phytoplasma australiense” strain strawberry lethal yellows phytoplasma NZSb11 [NC_021236.1]), CPsol_284/09 (“*Ca*. Phytoplasma solani” strain 284/09 [FO393427.1]), CPast_WBD (“Ca. Phytoplasma asteris” strain wheat blue dwarf [AVAO00000000.1]), CPpru_LHCF (“*Ca*. Phytoplasma pruni” strain CX [LHCF00000000.1]), and CPast_CYP (“*Ca*. Phytoplasma asteris” chrysanthemum yellows strain CYP [JSWH00000000.1]). CPast_AYWB (“*Ca*. Phytoplasma asteris” strain aster yellows witches broom [NC_007716.1]), CPpho_SA213 (“*Ca*. Phytoplasma phoenicum” [JPSQ00000000.1]) without *recA*, and CPho_A4 (AF515636), CPast_MBS (“*Ca*. Phytoplasma asteris” strain maize bushy stunt [NZ_CP015149.1]), and ROLP_MIEP (rice orange leaf phytoplasma LD1 [MIEP00000000.1]). Apalm_J233 *(Acholeplasma palmae* [NC_022538]*)* was used as an outgroup. “*Candidatus* Phytoplasma palmicola” is represented by one isolate (MZ11-005) from the 16SrXXII-A subgroup CPpml_XXII-A and by one isolate (GH04-009) from the 16SrXXII-B subgroup CPpml_XXII-B. Node values represent bootstrap test results with 500 replicates.

Seven housekeeping genes (*dnaC*, *gyrB*, *leuS*, *lpd*, *secA*, *rsmI*, and *rplV*) and 16S rRNA gene were found for 16 of 23 phytoplasma genomes or draft genomes. The 16S rRNA sequences of the corresponding phytoplasmas were used to construct a maximum likelihood (ML) phylogenetic tree after removal of a recombinant region of 155 bp. The ML phylogenetic tree presents a topology apparently similar to that of the housekeeping genes but increased the strength of the phylogenetic tree (Fig. 2). However, the congruency index (*I*_cong_) of 1.1039 was not significant (*P* = 0.35), indicating that the two calculated trees were not more congruent than what is expected by chance (46).

## DISCUSSION

### Multilocus sequence analysis highlights a genetic structure congruent with 16S rRNA subgroups.

To date, molecular characterization of “*Ca*. Phytoplasma palmicola” has been based mainly on the amplification of the 16S-23S rRNA gene operon by using the universal phytoplasma 16S rRNA gene primers, P1/P7 (47, 48), or specific primers (49), or, alternatively, on the amplification of the ribosomal protein gene (50) or the *secA* gene (51, 52). The MLST scheme we report is based on eight housekeeping genes and allows the deciphering of “*Ca*. Phytoplasma palmicola” genetic diversity as it could be applied to all CLYD-infected coconut samples from the different countries of origin and sampling dates. The protocol has been optimized to avoid a nested-PCR step, thereby reducing the risk of false positives. Because of their specificity for “*Ca*. Phytoplasma palmicola” and their efficiency in amplifying our entire sample set, each of the seven primer pairs developed in this study could be used independently to confirm “*Ca*. Phytoplasma palmicola” diagnostically.

Intraspecific diversity among *Mollicutes* observed by MLST is variable, from very low for Mycoplasma mycoides subsp. *mycoides* (53, 54) to high for Mycoplasma agalactiae and Mycoplasma bovis (18). Even though MLST is available for some phytoplasmas, it has not yet been applied on a large scale for phytoplasmas (55, 56). Although the present MLSA scheme allowed the differentiation of only 8 STs of “*Ca*. Phytoplasma palmicola,” it clearly differentiated three distinct populations distributed into two existing 16SrXXII subgroups. The current division of “*Ca*. Phytoplasma palmicola” into two distinct 16SrXXII subgroups (37) is consistent with the MLSA data, because the two distinct subgroups correspond to the lowest similarity value of the concatenated sequence of 96.21 to 96.25%, depending on the ST considered. The 16S rRNA sequences do not allow differentiation between the populations from Mozambique and Nigeria (16SrXXII-A), while MLSA shows a dissimilarity of 1.4%. The complementarity of the two methods, 16S rRNA sequencing and MLSA, for assigning strains to species or lineages has been described previously (57).

### “*Ca*. Phytoplasma palmicola” is subject to bottlenecks leading to a strong geographic structure.

Intracountry diversity of “*Ca*. Phytoplasma palmicola” measured by MLST is low, with the detection of 4 and 3 STs in Ghana and Mozambique, respectively. However, the identification of 3 STs in Mozambique contrasts drastically with the findings of Bila et al. (42), who observed high diversity and expanding populations of “*Ca*. Phytoplasma palmicola” in Mozambique based on both the *secA* and 16S rRNA genes, whereas those genes appear to be the most conserved in this study.

Except for the rare ST7, we observed four genetically related STs in Ghana versus two STs in Mozambique. A unique ST8 was observed in Nigeria, the first African country in which the disease was described, in 1917 (30). While only four Nigerian samples could be found and analyzed in this study, those samples were collected from coconuts separated by distances of up to 300 km. This contrasts to the situation in Ghana, where three or four STs were observed at similar distances. Only genotyping of a larger set of samples can confirm the lack of diversity in Nigeria.

The occurrence of strong bottlenecks might explain the very low diversity at the national or regional scales. Coconut lethal yellowing phytoplasmas lead to the rapid and inescapable death of the infected coconut in about 1 year. A new genetic phytoplasma variant has a short span of time to appear and emerge from the initial plant host. If such a variant appears, the probability for it to be acquired by the insect vectors would be low compared to that of the prevalent original genotype, unless this new variant acquires a higher multiplicative capacity in the coconut or a greater ability to be acquired by the insect vector.

### Geographic isolation of “*Ca*. Phytoplasma palmicola” puts potential intraspecific recombination at a disadvantage.

Phytoplasmas are considered to have a high level of plasticity through genome rearrangement and recombination (58–60), and both *recA*, when present, and PMU (potential mobile unit) genes would play a role in adapting to different environments (61, 62). Despite the presence of the *recA* gene in “*Ca*. Phytoplasma palmicola,” no signs of recombination were observed among its different populations. Since multiple infections have been observed for “*Ca*. Phytoplasma mali” (63), spatial aggregation of the different “*Ca*. Phytoplasma palmicola” STs and low diversity at the regional level could explain why we detected neither coinfection nor recombination. This distribution reduces the probability that two STs would be in the same plant and hence reduces the probability of observing signals of recombination. Detecting the occurrence of such a rare event of intraspecific recombination would require extensive sampling in places where genetically distant STs share the same geographic area, for example, in the Zambezia province in Mozambique with ST6 and ST7.

A second phytoplasma that infects coconut trees in Africa and that is responsible for lethal decline (LD) in Tanzania, “*Ca*. Phytoplasma cocostanzania,” has been described in both the Cabo Delgado (36) and Zambezia (42) provinces of Mozambique, where “*Ca*. Phytoplasma palmicola” is present. Interspecific coinfection has not yet been reported. The seven new primer pairs developed for the “*Ca*. Phytoplasma palmicola” MLST scheme were designed to be specific for “*Ca*. Phytoplasma palmicola,” and they are therefore not appropriate for the detection of interspecific coinfections. However, interspecific coinfection could not be detected through 16S rRNA or *rplV* gene amplification and sequencing, while the corresponding primers can detect both phytoplasmas (50, 64).

### New clues about “*Ca*. Phytoplasma palmicola” epidemiology.

Genotyping old historical samples would have been the best way to redraw the routes of introduction and the evolution of “*Ca*. Phytoplasma palmicola.” However, since phytoplasmas cannot be cultivated and since coconut cannot be grafted, historical “*Ca*. Phytoplasma palmicola” strains have not been maintained. Until now, we have been unable to find old frozen CLYD plant material or nucleic acids.

“*Ca*. Phytoplasma palmicola” STs are geographically clustered and distributed as small or large foci, suggesting a gradual spread. This observation is consistent with the previous observation of aggregated disease patterns and predominant dissemination within short distances (65). A low dispersion capacity of the unidentified “*Ca*. Phytoplasma palmicola” insect vector or poor acquisition or transmission efficiencies could explain this clustered geographic pattern. A longer-distance dissemination pattern would imply extreme weather events or human-assisted long-distance movements of nut, seedling, alternative plant hosts, or plant-carrying insect vectors.

Regarding alternative plant hosts for palm phytoplasmas, Bahder et al. (66) present the hypothesis that “*Ca*. Phytoplasma palmae” (group 16SrIV) could have been introduced in one plot in Florida by the planting of St. Augusta grass, on which *Haplaxus crudus*, identified as an insect vector of this phytoplasma, could achieve part of its life cycle as it does on many grasses (67). Studies are conflicting about possible “*Ca*. Phytoplasma palmicola” alternative plant hosts. No alternative hosts were identified in Ghana (68), whereas “*Ca*. Phytoplasma palmicola”-related strains were detected in different weed plant families in Ivory Coast (69). Surveys must be undertaken in other African countries to determine if these new findings are or are not restricted to this particular strain of “*Ca*. Phytoplasma palmicola” and to evaluate the potential role of these plant species as reservoirs for the short- and long-distance propagation of the disease. “*Ca*. Phytoplasma palmicola” presents a strong geographical pattern, in contrast to that of other phytoplasmas, such as fruit tree phytoplasmas in Europe (19). In fruit trees, both grafting and vegetative multiplication enhance dissemination and genetic uniformity; however, these practices do not exist for coconut trees. In general, human dissemination of coconut varieties is achieved through nuts or seedlings disseminated at the local or regional level. Dissemination at the continental or intercontinental scale is achieved through nuts or, more recently, coconut embryos. Phytoplasmas have been detected in coconut embryos (70, 71), and transmission to the seedling has been observed through the *in vitro* germination of embryos (72). However, the natural germination of phytoplasma-contaminated seeds and transmission of the phytoplasma to a viable seedling have not yet been demonstrated. Seed transmission may theoretically explain the occurrence of “*Ca*. Phytoplasma palmicola” of the subgroup 16SrXXII-A in both eastern and western Africa, because some nuts may have been introduced from West to East Africa (73). However, since at least 60 SNPs differentiate Nigerian and Ghanaian “*Ca*. Phytoplasma palmicola” strains from those detected in Mozambique, this excludes the recent introduction in Mozambique of a strain originating from areas we sampled in West Africa. It cannot be ruled out that the three genetically distinct African populations of “*Ca*. Phytoplasma palmicola” might have emerged independently due to a local plant host shift of an unidentified African phytoplasma. However, our data do not allow us to confirm such a possibility.

In Mozambique, ST7 is represented by a single sample and differs by at least 8 SNPs from the other Mozambican “*Ca*. Phytoplasma palmicola” STs. It is presently unknown whether several distinct introductions or a single introduction, followed by a local diversification, occurred in this country.

### “*Ca*. Phytoplasma palmicola” genetic diversity has implications for LYD management.

The only method for the large-scale control of LYD is early eradication (74–76) and/or deployment of genetic resistance (77, 78). During the last decades, many trials have been performed for the identification of promising coconut varieties with resistance to CLYD in Ghana (78), Mozambique, or, more recently, Ivory Coast, where resistance trials are ongoing. In Ghana, trials that were conducted in different locations and coconut varieties showed different disease levels between trials in Agona Junction and in Axim, where ST2 and ST1 are present, respectively (78). Geographical differences in disease incidence could be explained by differences in the aggressiveness or the epidemic propensity of the considered ST. Trials aimed to improve coconut germplasm must include the precise identification of the strain and a multisite establishment.

### An MLST scheme for “*Ca*. Phytoplasma palmicola”: a first step toward improved control of CLYD epidemics.

The proposed MLST scheme will enhance the resolution for better tracking of CLYD epidemics at the continental, regional, or provincial level. It could be implemented to identify the “*Ca*. Phytoplasma palmicola” strain that recently emerged in the Mozambican province of Inhambane. It could also confirm or refute the recently suggested introduction of “*Ca*. Phytoplasma palmicola” in Ivory Coast from western Ghana (41).

The development of an expanded MLST (eMLST) by including genes involved in the pathogenicity and insect transmission to the present MLST could increase its resolution. Using eMLST that had been expanded by adding two putative virulence genes (*ureG* and mba-np1) improved the discrimination of Ureaplasma urealyticum strains (79). Phytoplasma genes coding for surface proteins under positive selection, such as AMP/STAMP, IMP, or VMP, which were proven to participate in various steps of insect vector colonization, are particularly effective in phytoplasma molecular epidemiology (80–87). Only an IMP homolog was found to be encoded in the “*Ca*. Phytoplasma palmicola” genome draft. Such genes evolve faster than housekeeping genes and therefore do not fulfill the requirement defined by Maiden for the MLST scheme (2). The *imp* gene will be evaluated in a new eMLST scheme in further investigations.

On a smaller scale, MLST is clearly not sufficiently discriminating to trace “*Ca*. Phytoplasma palmicola” populations on a local or field scale. VNTRs and single nucleotide repeats (SNRs) are widely used to investigate monomorphic or poorly diversified bacterial species, such as Bacillus anthracis (88). If plastic *Mollicutes* genomes harbor large quantities of DNA repeats (89), their types and distributions are variable among phytoplasmas (11). Investigating the occurrence of VNTRs in phytoplasmas revealed that the repetition of short motifs is overrepresented in phytoplasmas compared to other prokaryotes, while long motifs are underrepresented (90). Although difficult to achieve, the identification of VNTRs applicable for inoculum tracing would benefit from a larger number of sequenced phytoplasma genomes. The ultimate approach is whole-genome sequencing (WGS). WGS of maize bushy stunt phytoplasma isolates from the same field successfully revealed genetic polymorphisms associated with functional traits (9).

In conclusion, the current MLST scheme will be valuable for assigning future outbreaks to existing or presently unreported genetic clusters of “*Ca*. Phytoplasma palmicola.” The interspecific comparisons presented in the present paper were based on available draft genomes. Generalization of this MLST scheme would require new genome sequencing to develop new sets of primers for each group or “*Candidatus* Phytoplasma” species. However, it would be a pertinent approach to compare the genetic diversities and to identify evolutionary constraints acting on the small genome of these insect-transmitted plant pathogens.

## MATERIALS AND METHODS

### Housekeeping gene selection and primer design.

Highly fragmented preliminary genome drafts obtained for one Mozambican isolate of the “*Ca*. Phytoplasma palmicola” subgroup 16SrXXII-A and one Ghanaian isolate of the “*Ca*. Phytoplasma palmicola” subgroup 16SrXXII-B (unpublished data) were used to select housekeeping genes and to design the corresponding PCR primers.

Housekeeping genes were selected for their availabilities on both drafts and their locations on different contigs to avoid contiguity. The two sequences of each selected gene from the two “*Ca*. Phytoplasma palmicola” genome drafts were aligned with BioEdit 7.0 software (http://www.mbio.ncsu.edu/BioEdit/bioedit.html) to produce a consensus sequence. Consensus sequences served as the templates to define specific PCR primers with predicted product sizes of 450 to 850 bp using Primer-BLAST (https://www.ncbi.nlm.nih.gov/tools/primer-blast). The seven housekeeping genes selected, primer sequences, annealing temperature, and PCR product sizes are shown in Table 1. An eighth gene, *rplV*, coding for the 50S ribosomal protein L22, was included in the MLSA scheme because of its capacity to differentiate two strains in Ghana (43).

### DNA phytoplasma: origin of the samples and DNA extraction.

In West Africa, Ghana and Nigeria are the only countries where the CLYD epidemics associated with “*Ca*. Phytoplasma palmicola” were active during the period of sampling (2004 to 2013). Sampling was extensively performed in Ghana during the 2004-2009 period, as the CLYD epidemics were very active. There was no restriction of sampling in any country. Ghana and Nigeria were subjected to extensive surveys in 2009 and 2012, respectively. In Nigeria, where the disease was first reported in 1932, four samples, collected in four different areas, were found positive for “*Ca*. Phytoplasma palmicola.” In Ivory Coast, “*Ca*. Phytoplasma palmicola” had not yet been reported at the time of the survey. A survey performed in Togo in 2006 was unsuccessful, and the disease was not reported during the 2004-2013 period. Similar feedback was obtained from Cameroon. In East Africa, LYTS is associated with “*Ca*. Phytoplasma cocostanzania” except in Mozambique, where both “*Ca*. Phytoplasma palmicola” and “*Ca*. Phytoplasma cocostanzania” are associated with distinct disease cases. In Mozambique, a survey in Cabo Delgado Province was done proposedly with systematic sampling of symptomatic coconut trees. Samples from the Zambezia Province of Mozambique were collected during a time-limited survey.

The MLST scheme was applied to the 132 “*Ca*. Phytoplasma palmicola” DNA samples (Table 4; Data Set S1 in the supplemental material). The DNA samples were extracted from stem sawdust or inflorescences of LYD-infected coconuts collected between 2004 and 2013 in Ghana (96 samples), Nigeria (4 samples), and Mozambique (32 samples). DNA was extracted using a modified CTAB protocol (39) or the DNeasy plant mini kit (Qiagen, Hilden, Germany).

**TABLE 4:** Origin of “*Ca*. Phytoplasma palmicola”-infected coconut trees analyzed

Country and region or province	No. of coconut trees sampled by yr								Total no.
	2004	2005	2006	2007	2009	2011	2012	2013	
Ghana	2	5	6	11	72				96
Central region		3	2	8	39				52
Volta region			3		16				19
Western region	2	2	1	3	17				25
Mozambique						13	4	15	32
Cabo Delgado								15	15
Zambezia						13	4		17
Nigeria							4		4
Total	2	5	6	11	72	13	8	15	132

### Housekeeping genes and 16S rRNA gene amplification and sequencing.

PCR amplification was performed for each of the seven selected housekeeping genes (i.e., genes encoding DNA recombinase A [*recA*], DNA gyrase subunit B [*gyrB*], leucyl-tRNA synthetase [*leuS*], dihydrolipoyl dehydrogenase [*lpd*], DNA replication protein [*dnaC*], protein translocase subunit [*secA*], and rRNA small subunit methyltransferase I [*rsmI*]) plus the *rplV* gene for the 132 isolates. The *rplV* gene was amplified with the rpLYF/rpLYR primer pair (50). In a subset of 41 “*Ca*. Phytoplasma palmicola” samples, the 16S rRNA gene was amplified for comparison. Amplification of the locus sequences was obtained by a single PCR performed in 25 μl containing a final concentration of 1× PCR buffer, 1.5 mM MgCl_2_, 0.2 mM each deoxynucleoside triphosphate, 0.25 μM each forward and reverse primer, and 1.25 U of GoTaq Flexi DNA polymerase (Promega, Madison, WI, USA). The 16S rRNA gene was amplified with the classic P1/P7 (48) primers. Both phytoplasma-free coconut tree DNA and water controls were systematically included as negative controls in all tests. Amplifications were performed with the GeneAmpPCR system 9700 (Thermo Fisher Scientific, Waltham, MA, USA) with an initial denaturation at 95°C for 2 min, followed by 35 cycles of 30 s at 94°C, 50 s at the optimal annealing temperature (*T_a_*) presented in Table 1, 60 s at 72°C, and a final 6-min extension at 72°C. For P1/P7 amplification, the annealing temperature was 58°C for 50 s with an extension step of 1 min 30 s at 72°C. The quality and concentrations of the amplified products were estimated on 1% agarose gels. PCR primers were used directly for double-strand sequencing of the PCR products by Genewiz (Takeley, United Kingdom). Two internal primers were designed for each strand to complete the P1/P7 sequencing.

Forward and reverse chromatograms were assembled using Geneious R8 (Biomatters Ltd., Auckland, New Zealand), extremities were trimmed to identical lengths and to start at the first base of a codon, and finally consensus sequences were exported for phylogenetic analyses. Because rpLYF/rpLYR primers amplify both the *rplV* sequence coding for the 50S ribosomal protein L22 and the *rpsC* genes coding for the 30S ribosomal protein, S3, the sequences were trimmed to eliminate the short intergenic and *rpsC* regions. The P1/P7 16S rRNA sequences were trimmed to eliminate the ITS1-tRNA-Ile-ITS2 and 23S rRNA gene sequences.

### “*Ca*. Phytoplasma palmicola” diversity analysis.

The number of polymorphic sites, nucleotide diversity (π), and average percent cGC were calculated by using DnaSP version 6.10. The *K_a_*/*K_s_* ratio, measuring the level of selection based on the ratio of the number of nonsynonymous substitutions per nonsynonymous site (*K_a_*) to the number of synonymous substitutions per synonymous site (*K_s_*), was calculated for each housekeeping gene by using MEGA version 7.0.26 (https://www.megasoftware.net). An integer neighbor-joining network (reticulation = 0.5) of the 132 concatenated sequences was constructed using PopART v1.7 software (91).

### Split network and recombination analysis.

A split network of the sequence types observed for each housekeeping gene was generated by using the neighbor-net method (92) of SplitTree4 v4.14 (93), and recombination was tested for each gene and for the concatenated sequences by performing the pairwise homoplasy index (*phi*) test (94). Recombination was also estimated for concatenated sequences by RDP4 (95).

### Interspecific analysis.

To conduct interspecific analysis, a total of 23 full and incomplete phytoplasma chromosomes available in GenBank were screened for the availability of all eight MLST-selected housekeeping genes and the 16SrRNA operon. Since some of the drafts lack complete *recA* sequences, the analysis was restricted to the seven other housekeeping genes (i.e., *dnaC*, *gyrB*, *leuS*, *lpd*, *secA*, *rsmI*, and *rplV*).

The data set included “*Ca*. Phytoplasma asteris” strain onion yellows OY-M (NC_005303.2), wheat blue dwarf (AVAO00000000.1), chrysanthemum yellows strain CYP (JSWH00000000.1), rice orange leaf phytoplasma (MIEP00000000.1), maize bushy stunt (NZ_CP015149.1), and aster yellow witches broom (NC_007716.1), “*Ca*. Phytoplasma mali” strain AT (CU469464.1); “*Ca*. Phytoplasma australiense” strains Australian grapevine yellows (NC_010544.1) and strawberry lethal yellows NZSb11 from New Zealand (NC_021236.1); “*Ca*. Phytoplasma pruni” strains milkweed yellows-MW1 (AKIL00000000.1), Italian clover phyllody MA1 (AKIM00000000.1), and vaccinium witches'-broom VAC (AKIN00000000.1); 16SrII-A phytoplasma strain peanut witches'-broom phytoplasma NTU2011 (AMWZ00000000.1); “*Ca*. Phytoplasma solani” strain 284/09 (FO393427.1); “*Ca*. Phytoplasma pruni” (LHCF00000000.1); and “*Ca*. Phytoplasma phoenicum” (JPSQ00000000.1). Acholeplasma palmae (NC_022538) was used as an outgroup.

The seven housekeeping gene sequences were aligned using Muscle (Codon) implemented in MEGA version 7.0.26 and then concatenated. The maximum likelihood (ML) phylogenetic tree was constructed by using the best-fitting model option of RDP4 (95), with recombinant regions removed when detected by more than three methods. Bootstrap values were calculated from 1,000 replications. The analysis was repeated with one of the two 16Sr RNA gene sequences of each genome. The congruence of the phylogenetic trees from the two sets of sequences (MLSA and 16S) were calculated by using the congruency index *I*_cong_ (46) (http://max2.ese.u-psud.fr/icong/index.help.html).

### Accession number(s).

The 1,056 sequences obtained are available in the European Nucleotide Archive (ENA) (https://www.ebi.ac.uk/ena/) under project no. PRJEB27044 (study ERP109081). The sequences can be found under accession numbers LR028084 to LR029139. The 16S rRNA sequences were deposited in the ENA under project no. PRJEB27044 with accession numbers LR028043 to LR028083.

## Supplementary Material

Supplemental file 1

Supplemental file 2
