# Supplementary material for: Multilocus Sequence Analysis Reveals Three Distinct Populations of “Candidatus Phytoplasma palmicola” with a Specific Geographical Distribution on the African Continent
Source: Appl Environ Microbiol. 2019 Apr 4;85(8):e02716-18. doi: 10.1128/AEM.02716-18 (PMC6450020; doi:10.1128/AEM.02716-18)
Supplement: Supplemental file 1 [file AEM.02716-18-s0001.pdf]

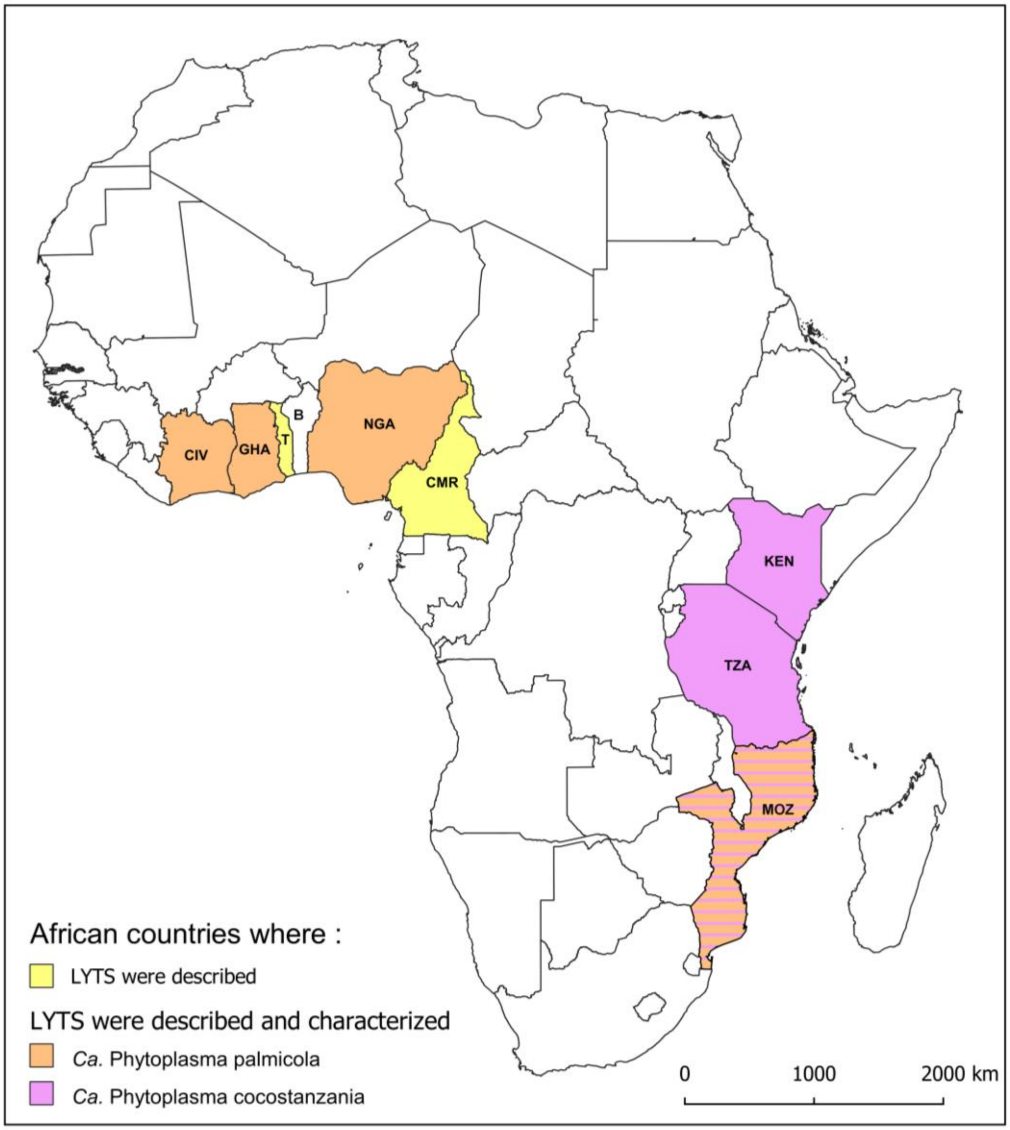

Figure S1: Distribution of Lethal Yellowing Type Syndromes (LYTS) in Africa. From west to east, LYTS were described in Ivory Coast (**CIV**) (1), Ghana (**GHA**) (2), Togo (**T**:**TGO**) (3), Nigeria (**NGA**) (4), Cameroon (**CMR**) (5), Kenya (**KEN**) (6), Tanzania (**TZA**) (7) and Mozambique (**MOZ**) (8). '*Candidatus* Phytoplasma palmicola' (9) is responsible for LYTS in Ghana and Nigeria (10), in Ivory Coast (11), and in Mozambique (12). '*Candidatus* Phytoplasma cocostanzania' (13) was identified in Kenya and Tanzania (10, 12), and in the north of Mozambique (14). Phytoplasmas responsible for LYTS in countries presented with yellow color have never been characterized. LYTS have never been reported in Benin (**B**:**BEN**).

Reference list Figure S1

1. Konan Konan J, Allou K, Atta Diallo H, Saraka Yao D, Koua B, Kouassi N, Benabid R, Michelutti R, Scott J, Arocha-Rosete Y. 2013. First report on the molecular identification of the phytoplasma associated with a lethal yellowing-type disease of coconut palms in Cote d'Ivoire. New Disease Reports 28:3.
2. Chona BL, Addoh PG. 1970. Cape St. Paul wilt: the present position, p 3-8, Coconut in Ghana Bulletin no 3, Chona, B.L. and Adansi, M.A. ed. Crops Research Institute. Council for Scientific and Industrial Research (Ghana).
3. Meiffren M. 1951. Note préliminaire sur l'étude de la maladie des cocotiers au Togo. Agronomie tropicale 6:163-174.
4. Johnson WH. 1918. Annual report of the agricultural department of southern provinces Nigeria for the year 1917, Ibadan, 25th. Government Publication:14 p.
5. Grimaldi J, Monveiller G. 1965. La Maladie des cocotiers de Kribi. Cameroun agricole pastoral et forestier 90:18-27.
6. Dowson W. 1921. Some problems of economic biology in East Africa (Kenya Colony). Annals Applied Biology 8:85-87.
7. Stein H. 1905. Die Kokosnuss und deren Bearbeitung in Deutsch-Ostafrika. Der Tropenpflanzer 9:195-201.
8. de Carvalho T, Mendes O. 1958. Doenças de plantas em Moçambique. Província de Moçambique, Direcção de agricultura e florestas, repartição de sanidade vegetal, secçaõ de patologia vegetal.
9. Harrison NA, Davis RE, Oropeza C, Helmick EE, Narváez M, Eden-Green S, Dollet M, Dickinson M. 2014. '*Candidatus* Phytoplasma palmicola', associated with a lethal yellowing-type disease of coconut (*Cocos nucifera* L.) in Mozambique. International Journal of Systematic and Evolutionary Microbiology 64:1890-1899.
10. Tymon AM, Jones P, Harrison NA. 1998. Phylogenetic relationships of coconut phytoplasmas and the development of specific oligonucleotide PCR primers. Annals of Applied Biology 132:437-452.
11. Arocha-Rosete Y, Konan Konan JL, Diallo AH, Allou K, Scott JA. 2014. Identification and molecular characterization of the phytoplasma associated with a lethal yellowing-type disease of coconut in Côte d'Ivoire. Canadian Journal of Plant Pathology 36:141-150.
12. Mpunami, Tymon, Jones, Dickinson. 1999. Genetic diversity in the coconut lethal yellowing disease phytoplasmas of East Africa. Plant Pathology 48:109-114.
13. IRPCM phytoplasma/spiroplasma working group. 2004. '*Candidatus* Phytoplasma', a taxon for the wall-less, non-helical prokaryotes that colonize plant phloem and insects. International Journal of Systematic and Evolutionary Microbiology 54:1243-1255.
14. Dollet M, Lourenço E, Macome F, Vaz A, Fabre S. 2012. In Mozambique, at least two different phytoplasmas induce Lethal Yellowing Type Syndromes in coconut palms, Congress of the International Organization for Mycoplasmology, Toulouse, France, 2012-07-15 / 2012-07-20. s.n., public.

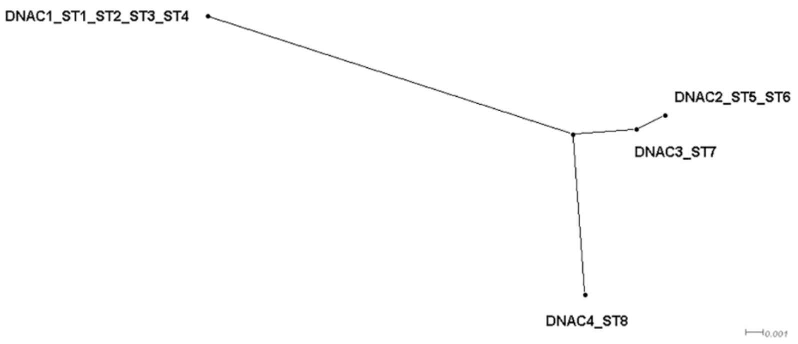
DNAC1_ST1_ST2_ST3_ST4
DNAC2_ST5_ST6
DNAC3_ST7
DNAC4_ST8
0.001

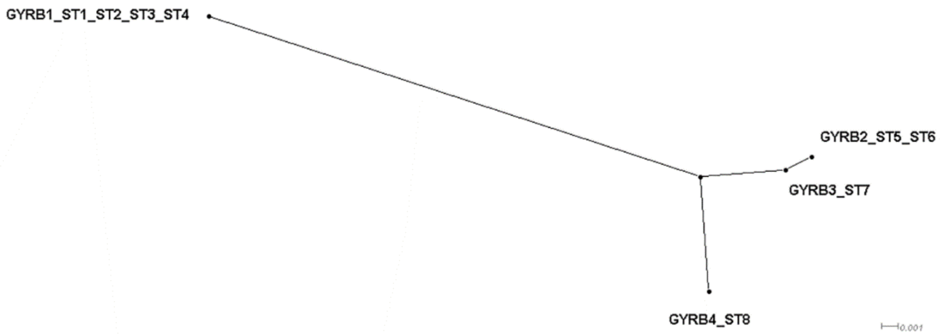
GYRB1_ST1_ST2_ST3_ST4
GYRB2_ST5_ST6
GYRB3_ST7
GYRB4_ST8
0.001

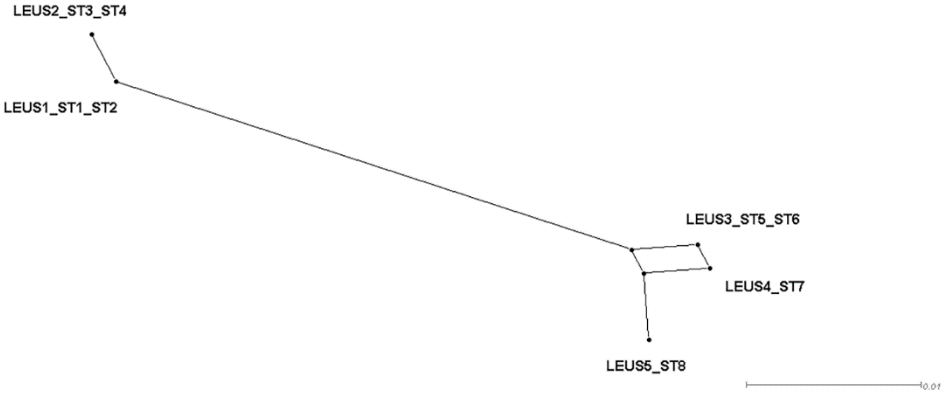
LEUS2_ST3_ST4
LEUS1_ST1_ST2
LEUS3_ST5_ST6
LEUS4_ST7
LEUS5_ST8
0.01

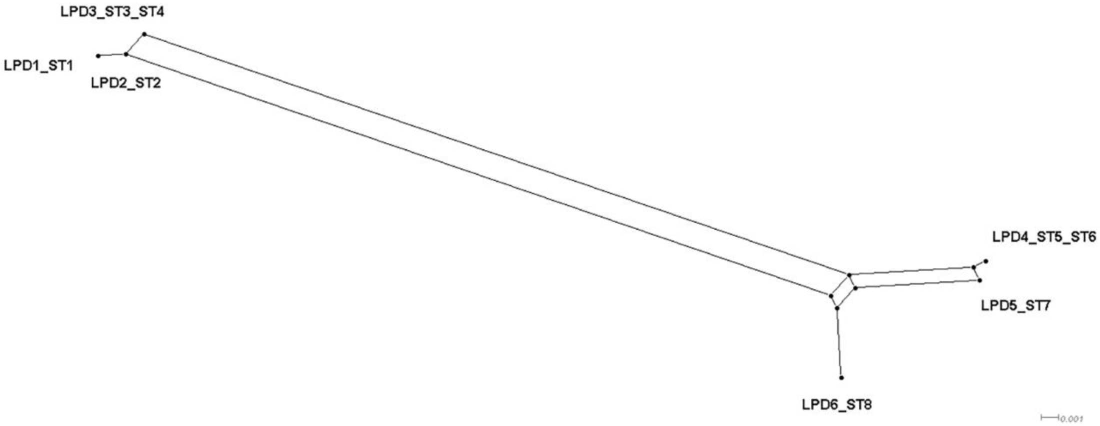
LPD3_ST3_ST4
LPD1_ST1
LPD2_ST2
LPD4_ST5_ST6
LPD5_ST7
LPD6_ST8
0.001

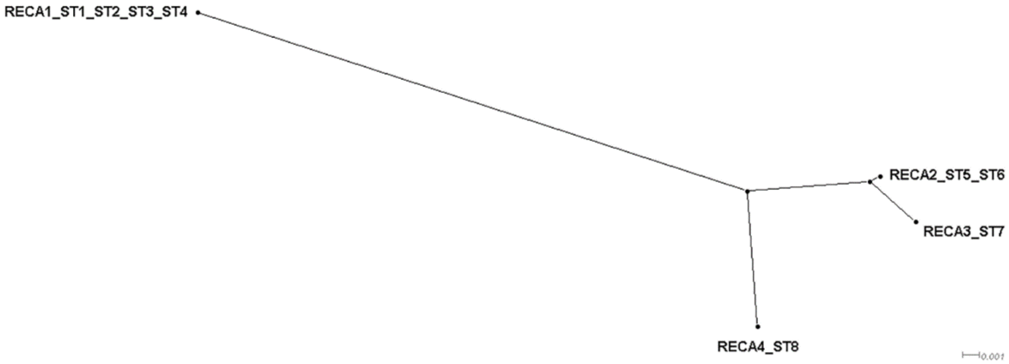
RECA1_ST1_ST2_ST3_ST4
RECA2_ST5_ST6
RECA3_ST7
RECA4_ST8
0.001

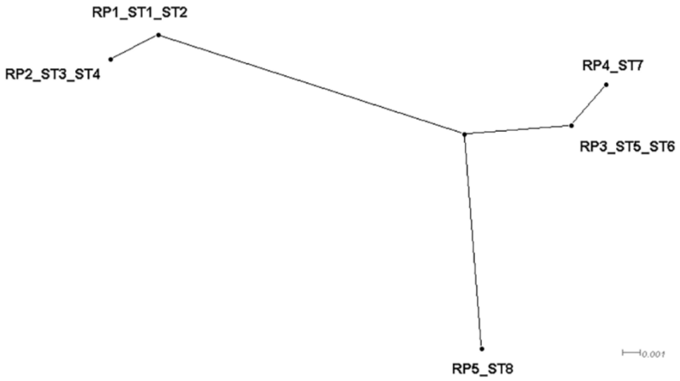
RP1_ST1_ST2
RP2_ST3_ST4
RP4_ST7
RP3_ST5_ST6
RP5_ST8
0.001

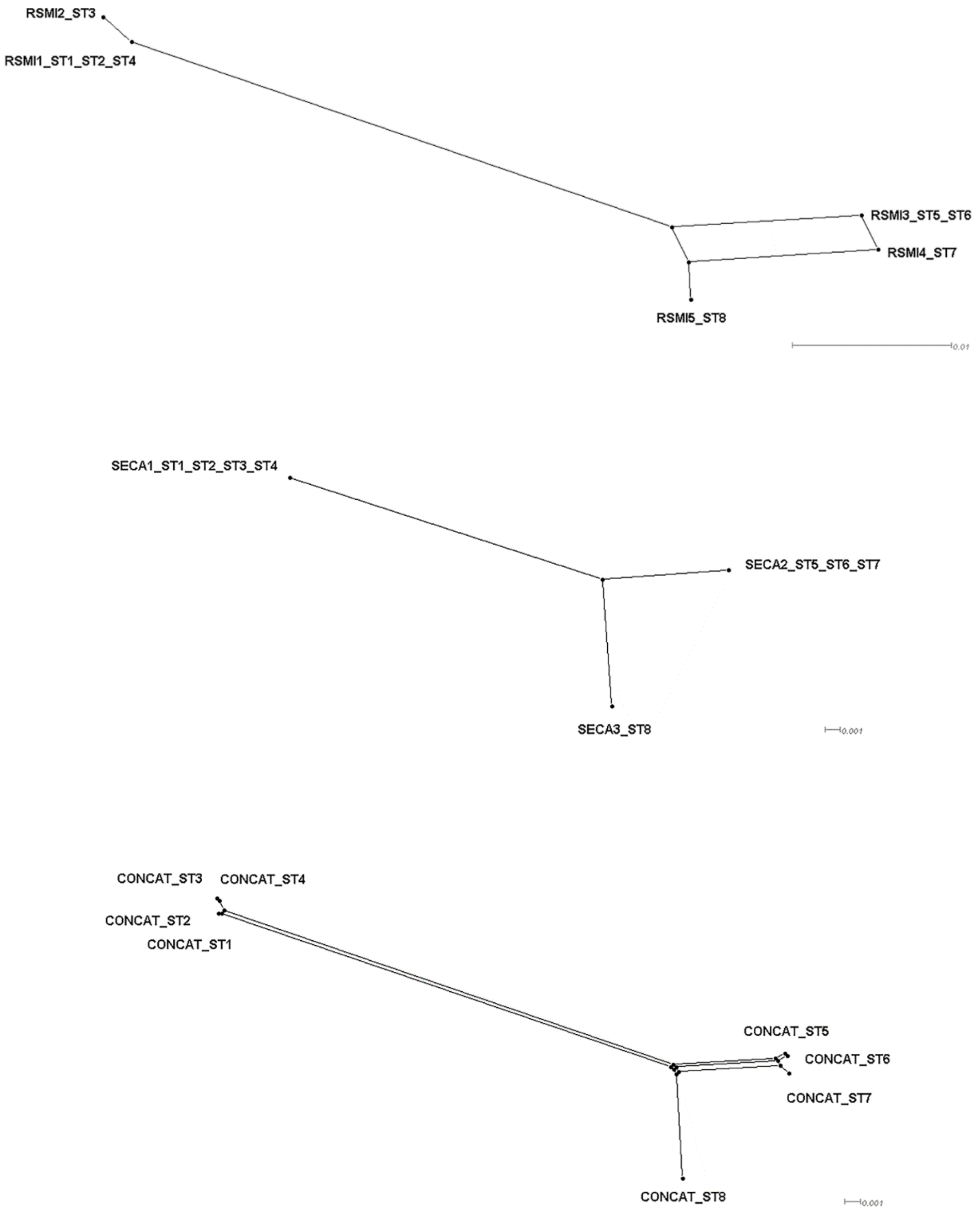

Figure S2. Split network from the alignments of the different *Candidatus* Phytoplasma palmicola sequence types for each of the 8 housekeeping genes (*dna*C, *gyr*B, *leu*S, *lpd*, *rec*A, *rpl*V, *rsm*I and *sec*A) and the concatenated sequences (*C*oncat) of the eight housekeeping genes.
